# Statins and new-onset atrial fibrillation in a cohort of patients with hypertension. Analysis of electronic health records, 2006–2015

**DOI:** 10.1371/journal.pone.0186972

**Published:** 2017-10-26

**Authors:** Lia Alves-Cabratosa, Maria García-Gil, Marc Comas-Cufí, Anna Ponjoan, Ruth Martí-Lluch, Dídac Parramon, Jordi Blanch, Marc Elosua-Bayes, Rafel Ramos

**Affiliations:** 1 Vascular Health Research Group of Girona (ISV-Girona). Institut Universitari d’Investigació en Atenció Primària Jordi Gol (IDIAP Jordi Gol), Girona, Catalonia, Spain; 2 Institut d’Investigació Biomèdica de Girona (IDIBGI), Dr. Josep Trueta University Hospital, Girona, Catalonia, Spain; 3 Translab Research Group. Department of Medical Sciences, School of Medicine, University of Girona, Girona, Catalonia, Spain; 4 Primary Care Services, Girona. Catalan Institute of Health (ICS), Girona, Catalonia, Spain; University of British Columbia, CANADA

## Abstract

Hypertension is the most prevalent risk factor for new-onset atrial fibrillation (AF). But few studies have addressed the effect of statins on the incidence of this arrhythmia in patients with hypertension. This study aimed to evaluate the effect of statins on new-onset of this arrhythmia in a hypertensive population, accounting for AF risk. Data from the Information System for the Development of Research in Primary Care was used to recruit a retrospective cohort of ≥55-year-old hypertensive individuals with no ischemic vascular disease, in 2006–2007, followed up through 2015. The effect of initiating statin treatment on new-onset atrial fibrillation was assessed with Cox proportional hazards models adjusted by the propensity score of receiving statin treatment, in the overall study population and stratified by AF risk. Of 100 276 included participants, 9814 initiated statin treatment. The AF incidence per 1000 person-years (95% confidence interval) was 12.5 (12.3–12.8). Statin use associated with a significant (9%) reduction in AF incidence. Differences in absolute AF incidence were higher in the highest AF risk subgroup, and the estimated number needed to treat to avoid one case was 720. The relative effect was poor, similar across groups, and non-significant, as was the association of statins with adverse effects. We found a limited protective effect of statins over new-onset AF in this hypertensive population with no ischemic vascular disease. If there is no further indication, hypertensive patients would not benefit from statin use solely for AF primary prevention.

## Introduction

Atrial fibrillation (AF) conveys a huge social, medical, and economic burden because it is the most common arrhythmia in clinical practice and it associates with quality of life detriment [1], and with increased mortality and morbidity risk, mainly from stroke and heart failure [2–5].

Clinical management of AF is based on strategies for rhythm and rate control, as well as thromboprophylaxis [2]. But this arrhythmia tends to worsen, leading to longer, more frequent attacks, and becoming a chronic condition because current approaches are limited [6,7]. Therefore, research on prevention strategies is strongly encouraged, including the potential protective effect of statins [8]. Beyond their lipid-lowering effect, statins have been hypothesized to target the electrical and structural transformation that constitutes the substrate for AF, atrial remodelling, without the concomitant pro-arrhythmic effect associated with the anti-arrhythmic drugs [9].

The effect of statins on new-onset AF has been analysed in a variety of populations [2,7,10]. A metaanalysis that compared studies with long versus short follow-up found no benefit of statins on atrial fibrillation in the trials with longer term follow-up [10]. But studies are scarce in patients with hypertension, which is the most prevalent risk factor for new-onset AF. The hypertensive population would have specific pathophysiological mechanisms [11], towards which prevention of new-onset AF could be directed. Additionally, the evaluation of AF risk could allow population stratification and aid in patient counselling [12–14]. Both the study of patients with hypertension and AF risk assessment could be useful to target individuals requiring intervention and tailor preventative approaches to tackle this arrhythmia [12,13,15].

The few studies that addressed the effect of statins on new-onset AF included people with ischemic heart disease [16], and thus, with statins as prophylaxis [17]. We found no studies on the association of statins with incident AF in the older hypertensive population without ischemic heart disease. Neither has the effect of statins on incident AF been analysed in the context of individual risk for this rhythm disturbance.

We examined the association of statins with incident AF in a hypertensive population without ischemic vascular disease, according to their risk of suffering this arrhythmia.

## Methods

### Data source

Data were obtained from the Information System for the Development of Research in Primary Care (SIDIAP^Q^) [18] database, which is a subset of SIDIAP optimised for research. SIDIAP contains longitudinal medical records of a representative sample of patients attended by general practitioners (GPs) in Catalonia, and covers about 80% of the total of 7.5 million persons of Catalonia, attended in the primary care practices managed by the Catalan Institute of Health [18]. SIDIAP data include demographic information, coded clinical diagnoses using the International Classification of Diseases 10^th^ revision (ICD-10), specialist referrals, hospital discharge information (ICD-9), laboratory tests, and treatments (drug prescriptions and corresponding pharmacy invoicing data). Encoding of identifiers ensures confidentiality of the information in the SIDIAP database. GPs follow regulated protocols on data recording, and are externally assessed for its completeness and continuity. Those records that are accredited to be accurate and complete over predefined data quality standards constitute SIDIAP^Q^ [19], with which the present study was carried out. SIDIAP^Q^ contains anonymised information on about 2 million patients, attended by 1365 GPs, yielding almost 20 million person-years for the period 2006–2015 [19], and it has been widely used in previous epidemiological research [20–24]. Ethics approval for research using SIDIAP^Q^ data was obtained from the Ethics Committee for Clinical Research IDIAP Jordi Gol (P14/052).

### Study population

We included patients aged 55 years or older, with previous hypertension without target organ involvement. Hypertension was defined with the codes I10 and I15 in ICD-10, 401 in ICD-9, or with any antihypertensive treatment (adrenergic beta-antagonists, diuretics, calcium channel blockers, agents acting on the renin-angiotensin system, or other antihypertensive drugs).

### Exclusion criteria

Patients were excluded if they had a history of AF, other arrhythmias, ischemic vascular disease (defined as ischemic heart disease, stroke, transient ischemic attack, or peripheral artery disease), any revascularization procedure, or a filled prescription for any of the following medications: antiarrhythmics (class I and III), selective calcium channel blockers with direct cardiac effects, digitalis, nitrates, and vitamin K antagonists, as surrogates of the exclusion conditions. We also excluded individuals without a score on MEDEA deprivation index [25].

To avoid frailty bias, we further excluded patients with cancer, dementia, plegia, who received a transplanted organ, or who were institutionalised, in dialysis, or under treatment for cardiac conditions (Anatomical Therapeutic Chemical Classification code C01) at baseline. Patients who had been under statin treatment before the entry date were also excluded, to avoid a potential indication bias.

### Study design and length of follow-up

We carried out an historical cohort study from July 2006 through December 2015, to analyse the effect of statin initiation on new-onset AF. Patients were defined as new-users if they had purchased statins for the first time during the recruitment period (from July 2006 through December 2007). New-users and non-users (controls) kept their status during follow-up, resembling the intention-to-treat approach in randomised controlled trials. Entry date was the day of the first statin purchase, for new-users who met inclusion criteria. Controls were ascribed a randomised entry date based on the distribution of new-users entry dates; they were excluded if inclusion criteria were no longer met at the ascribed entry date. Baseline period was defined as 1 year previous to entry date. Censoring applied to transfer from SIDIAP, end of study period, or death, whichever occurred first.

### Exposure

Statin initiation was the main exposure. Patients were considered highly adherent to statin treatment when their medication possession ratio (MPR) was ≥70%, over 6 months.

Participants were stratified according to different risks of suffering new-onset AF, as follows: less than 2.5% of new-onset AF risk at 5 years, ≥2.5 to <7.5%, and ≥ 7.5% [26]. The risk level was determined with a validated 5-year risk function of new-onset AF, developed in this hypertensive population without ischemic vascular disease (see Table A in S1 Appendix).

### Outcomes

We defined new-onset AF with the first entry of the following codes: I48 (ICD-10), and 4273 and subcategories (ICD-9). We also examined the adverse effects of being a statin user. Liver toxicity and myopathy were considered attributable to statins if they occurred within the first year of treatment. Diabetes, hemorrhagic stroke, and malignant neoplasms were considered attributable to statins if they occurred after one year of statin initiation: they were considered more likely to be associated with long-term statin use [27].

### Baseline covariates

The following covariates at baseline were considered potentially associated to statin treatment and to the study outcome: age, sex, a deprivation index (developed for Spain by the MEDEA researchers[25]), height, weight, systolic and diastolic blood pressure (BP), smoking, glucose, total cholesterol, high (HDL) and low (LDL) density lipoprotein cholesterol, obesity, dyslipidaemia, valvular heart disease, hypertension, diabetes, asthma, chronic obstructive pulmonary disease, sleep apnoea, arthritis, hyperthyroidism, hypothyroidism, chronic kidney disease, heart failure, treatments other than statins (non-statin lipid-lowering drugs, diuretics, beta blocking agents, calcium-channel blockers, agents acting on the renin-angiotensin system, other antihypertensives, antidiabetic drugs, corticosteroids for systemic use, anti-inflammatory and antirrheumatic drugs, psycholeptics, psychoanaleptics), and coronary heart disease risk according to the Framingham function adapted to the Spanish population, duly validated and named the Framingham-REGICOR risk function [28].

### Statistical methods

Continuous variables were expressed as mean (standard deviation) and categorical variables as percentages. We used 10 multiple imputations by chained equations [29] to replace the missing baseline values of systolic and diastolic BP, pulse pressure, weight, height, body mass index (BMI), glycaemia, total cholesterol, HDL and LDL cholesterol, and triglycerides. In sensitivity analyses, we compared results restricted to the population with complete data and those including imputed data (see Sensitivity Analyses in Tables C-E in S1 Appendix).

To avoid the selection bias associated with non-random treatment allocation, we derived a logistic model based on variables that could potentially influence the odds of receiving statin prescription, and obtained the propensity score (PS) of statin treatment for each study participant. Variables were considered well balanced if the standardised differences between new-users and non-users were <0.10 after adjusting by PS.

We built Cox proportional hazard models to estimate the effect of being a new user on new-onset AF, amongst all the population and within risk strata, adjusted by the PS of initiating statin treatment. In sensitivity analyses, we studied the subpopulation of patients with high adherence to statin treatment.

We also estimated the adverse effects of being a new user of statins. We tested linearity of the PS with respect to new-onset AF and the proportionality of hazards assumption. We also calculated crude incidences and 1-year number needed to treat (NNT) to prevent one additional case of new-onset AF, or to contribute to one additional case of the adverse effects considered. Statistical significance was set at p<0.01.

All analyses were conducted using R-software [30] (version 3.0.1; R Foundation for Statistical Computing, Vienna, Austria), including MICE package for multiple imputation [31].

## Results

### Study population

**Fig 1:**
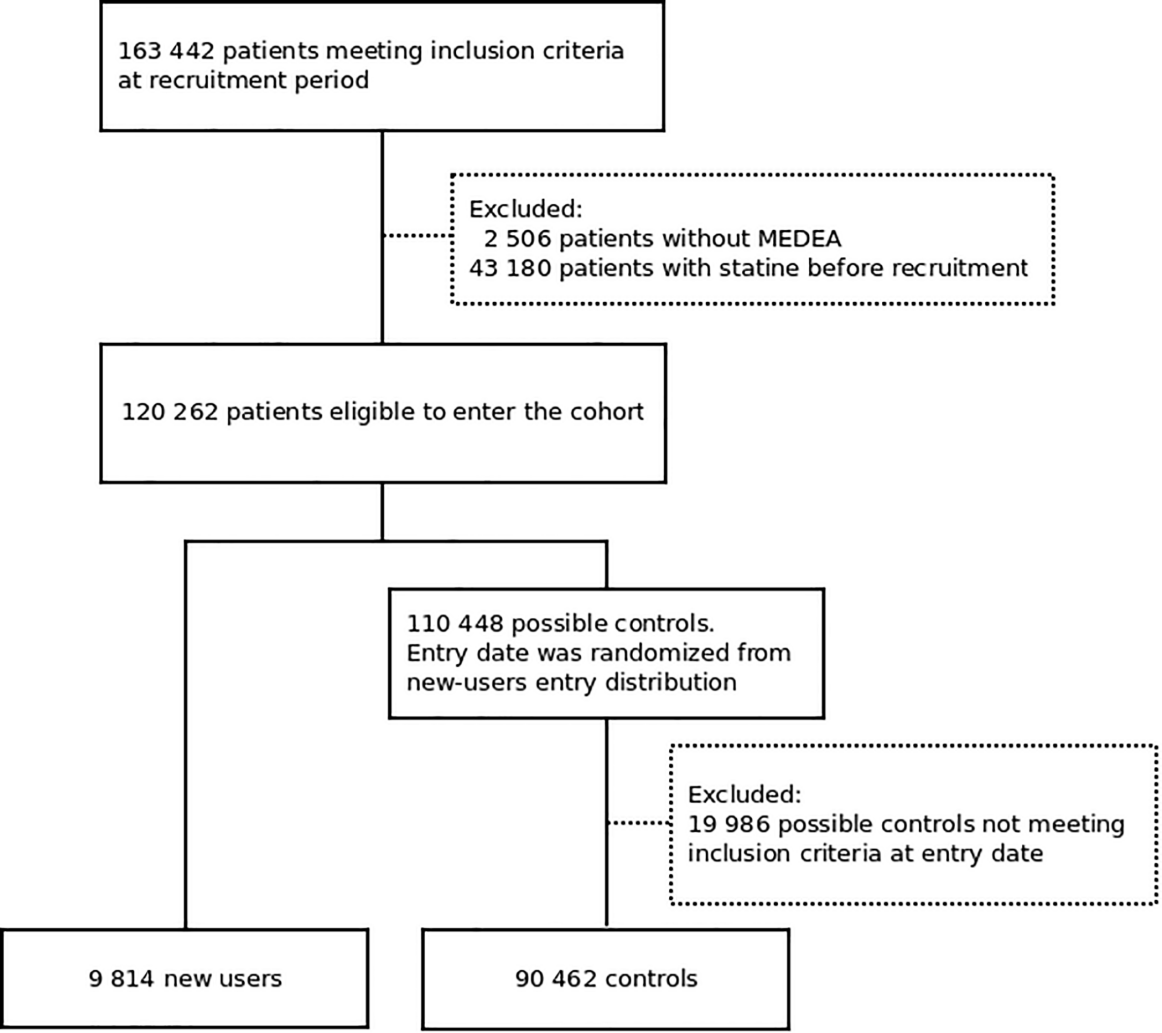
Study flowchart.

From July 2006 through December 2015, SIDIAP^Q^ recorded 163 442 eligible patients, of which 100 276 were included in the cohort and 9814 (9.8%) of these initiated statin treatment (Fig 1). Median (1^st^-3^rd^ quartile) MPR was 67% (33–100%), and 90% of new-users had high adherence to treatment (6-month MPR ≥70%). Median (1^st^-3^rd^ quartiles) follow-up was 8.5 years (8.1–8.9) (see Table B in S1 Appendix, which shows the follow-up by risk groups).

Table 1 displays the baseline characteristics of the study population, comparing new-users with controls, before and after adjusting by PS. Before PS adjustment, new-users had worse lipid profile, higher prevalence of diabetes, and slightly higher prevalences of comorbidities and concomitant treatments. Standardised differences were <0.10 after adjusting by PS.

**Table 1:** Baseline characteristics of new-users and non-users of statins before and after propensity score adjustment.

	Before PS adjustment			After PS adjustment		
	Statin new-users (n = 90462)	Non-users (n = 9814)	SDf	Statin new-users (n = 90462)	Non-users (n = 9814)	SDf
Age, years	67.3 (8.1)	68.2 (8.9)	0.11	68.2 (8.0)	68.1 (8.8)	-0.01
Men	39.0	40.2	0.02	39.6	40.1	0.01
MEDEA index						
Rural areas	14.4	17.8	0.09	17.7	17.1	-0.02
Urban areas (quintile)						
First	6.0	6.1	<0.01	5.9	6.1	0.01
Second	13.9	13.8	<0.01	13.5	13.8	0.01
Third	21.3	20.6	-0.02	20.4	20.7	0.01
Fourth	22.3	21.1	-0.03	21.1	21.3	<0.01
Fifth	22.0	20.6	-0.04	20.7	20.7	<0.01
Systolic BP, mmHg	138.5 (16.3)	137.6 (16.3)	-0.05	137.9 (16.3)	137.7 (16.3)	-0.01
Diastolic BP, mmHg	79.4 (9.5)	78.9 (9.5)	-0.06	79.0 (9.5)	78.9 (9.5)	-0.01
Pulse pressure	59.1 (14.4)	58.8 (14.6)	-0.02	58.9 (14.4)	58.8 (14.6)	-0.01
Weight, Kg	75.6 (13.4)	74.6 (13.4)	-0.08	74.5 (13.4)	74.7 (13.4)	0.02
Height, cm	158.7 (8.9)	158.9 (9.1)	0.02	158.8 (8.9)	158.9 (9.1)	0.01
BMI, Kg/m^2^	30.0 (4.8)	29.5 (4.8)	-0.10	29.5 (4.8)	29.5 (4.8)	0.01
Total cholesterol, mg/dl*	245.0 (40.5)	209.8 (32.6)	-0.96	213.5 (22.9)	213.2 (20.3)	-0.01
HDL-c, mg/dl	56.6 (14.0)	57.7 (14.2)	0.08	57.7 (14.0)	57.5 (14.2)	-0.01
LDL-c, mg/dl	159.3 (36.2)	129.1 (28.8)	-0.92	132.1 (22.4)	132.0 (18.6)	-0.01
Triglycerides, mg/dl	145.8 (84.9)	115.2 (57.3)	-0.42	120.7 (79.6)	118.0 (53.7)	-0.04
Glucose, mg/dl	110.2 (34.8)	102.4 (26.4)	-0.25	103.8 (34.1)	103.1 (25.9)	-0.02
Alcohol consumption						
None	93.4	95.0	0.07	93.9	95.0	0.05
Low-risk	6.0	4.5	-0.06	5.6	4.5	-0.05
High-risk	0.6	0.4	-0.03	0.5	0.4	-0.01
Smoking	19.5	17.9	-0.04	18.0	18.1	<0.01
Diabetes	22.3	13.8	-0.22	13.0	13.5	0.02
Arthritis	1.2	1.0	-0.02	1.0	1.0	<0.01
Hyperthyroidism	1.0	0.9	-0.01	0.9	0.9	<0.01
Hypothyroidism	5.5	4.5	-0.04	4.4	4.6	0.01
COPD	6.4	6.5	<0.01	6.6	6.5	<0.01
Asthma	4.2	4.1	<0.01	4.1	4.1	<0.01
Sleep apnoea	1.7	1.4	-0.03	1.3	1.4	<0.01
Chronic kidney disease	2.2	1.8	-0.03	1.8	1.8	<0.01
Valvular heart disease	1.8	1.6	-0.02	1.6	1.7	<0.01
Heart failure	0.9	0.9	<0.01	0.9	0.9	<0.01
Concomitant treatment						
Diuretics	33.3	27.1	-0.13	26.8	27.5	0.02
Beta blockers	15.3	12.4	-0.09	12.1	12.5	0.01
Calcium channel blockers*	13.0	9.7	-0.10	9.2	9.8	0.02
Agents acting on renin-angiotensin system	60.2	46.5	-0.28	45.5	48.0	0.05
Other antihypertensives	4.1	3.4	-0.04	3.3	3.5	0.01
Hypoglycemic Agents	17.8	9.0	-0.26	7.4	8.2	0.03
Lipid-lowering drugs, non-statins	5.1	2.4	-0.15	1.9	2.1	0.02
AF risk subgroups						
<2.5%	29.0	28.4	-0.01	28.0	28.5	0.01
≥2.5–7.5%	51.8	48.6	-0.06	50.0	48.8	-0.02
≥7.5%	19.2	23.0	0.09	21.7	22.6	0.02
Framingham-REGICOR risk †	6.7 (5.4)	5.2 (4.4)	-0.31	5.5 (5.3)	5.3 (4.3)	-0.05
Framingham-REGICOR <10% risk (subgroups,%) ‡						
AF risk <2.5%	88.4	96.3	0.30	96.6	96.9	0.02
AF risk ≥2.5–7.5%	78.9	89.1	0.28	89.7	89.7	<0.01
AF risk ≥7.5%	70.3	77.7	0.17	79.4	77.6	-0.04

Data are displayed as % or mean (SD).

* Selective calcium channel blockers with mainly vascular effects.

^†^ Framingham-REGICOR coronary risk function [28].

‡Among those with a score <10% on the Framingham-REGICOR coronary risk function [28], % of participants within each AF risk subgroup.

BMI indicates body mass index; BP, blood pressure; COPD, chronic obstructive pulmonary disease; HDL, high density lipoprotein; LDL, low density lipoprotein; MEDEA, socioeconomic deprivation index; N, number of cases; PS, propensity score of statin treatment; SD, standard deviation; SDf, standardised differences.

Missing data count and a comparison between the imputed and the complete case dataset are shown in Tables C and D in S1 Appendix. Mean values of the imputed variables tended to be lower, as expected (see Table C in S1 Appendix). Overall, the population in the complete case dataset had higher prevalence of diabetes and slightly higher prevalence of comorbidities and concomitant treatments; this pattern persisted after PS adjustment (Table D in S1 Appendix).

Overall, 9873 participants had a record of new-onset AF, a total crude incidence of 12.5 per 1000 person-years (95% confidence interval–CI-, 12.3–12.8). Table 2 shows the crude AF incidence was higher in non-users of statins.

**Table 2:** Hazard ratios of statin use for incident atrial fibrillation and adverse effects of statins.

	New-users		Non-users			
	Events	Incidence rate* (95% CI)	Events	Incidence rate* (95% CI)	HR (95%CI)	NNT+
AF, total population	834	10.6 (9.8–11.3)	9039	12.7 (12.5–13.0)	0.91 (0.84–0.99)	1366
***AF risk group***						
<2.5%	75	3.1 (2.4–3.9)	785	3.6 (3.3–3.9)	0.91 (0.69–1.21)	5884‡
≥2.5 to <7.5%	420	10.1 (9.1–11.2)	4117	11.6 (11.3–12.0)	0.97 (0.86–1.08)	4590‡
≥7.5%	338	25.3 (22.5–28.1)	4137	29.6 (28.6–30.5)	0.93 (0.82–1.06)	720‡
***Adverse effects***						
Cancer	1460	22.0 (20.8–23.1)	13463	22.4 (22.1–22.8)	1.03 (0.97–1.09)	-
Hemorrhagic stroke	126	1.8 (1.5–2.1)	1373	2.1 (2.0–2.2)	0.84 (0.69–1.03)	-
Diabetes	2094	34.6 (33.1–36.1)	15628	27.3 (26.9–27.7)	0.97 (0.92–1.02)	-
Hepatotoxicity	8	0.8 (0.2–1.4)	57	0.6 (0.5–0.8)	-	-
Myopathy	3	-	23	0.3 (0.1–0.4)	-	-

*per 1000 person-year.

+at 1 year.

‡estimated.

AF indicates atrial fibrillation; CI confidence interval; HR hazard ratio; NNT, number needed to treat.

### Effect of statins on AF incidence

Initiation of statin treatment associated with a significant reduction of AF incidence of 9%. Sensitivity analysis of AF incidence in patients with MPR ≥70% at 6 months showed a similar, although not significant, effect (HR 0.91; 95% CI 0.81–1.02). Table 2 shows the hazard ratios (HRs) for statin new-users and the statin NNT of incident AF for the whole study population and for each risk-level subgroup. The association of statins with AF incidence did not differ by risk subgroups, and the size effect was similar to that in the overall study population, although non-significant. Similar results are displayed in analyses restricted to the complete case dataset (see Table E in S1 Appendix). Table B in S1 Appendix shows predicted risk compared well with observed risk within each 5-year risk-level subgroup.

### Adverse effects of statins

We observed no significant association of statin initiation with its potential adverse effects (Table 2). Similar results of analysis restricted to the complete case dataset are displayed in Table E in S1 Appendix.

## Discussion

### Main findings

Statin initiation showed a minimal but significant association with lower AF incidence. The absolute risk reduction increased with higher estimated risk, whereas the relative risk of this arrhythmia was similar across risk stratification and did not reach statistical significance, probably because splitting the population into risk subgroups reduced statistical power. The 1-year NNT for new-onset AF was too high to advocate the use of statins in primary prevention of this arrhythmia, not even in the highest risk group. We found no association of statins with the studied adverse effects.

### Previous studies

Statins reduced the risk of new-onset AF less than what has been reported for other outcomes, such as secondary prevention of ischemic heart disease [32]. The 2014 AHA guidelines on AF reported no benefit of statins in primary prevention of this arrhythmia in patients without cardiovascular disease [7], and the latest ESC guidelines stated the lack of effect of statins in any setting [2,10].

With regard to patients with hypertension, the meta-analysis cited in these ESC guidelines [10] included two studies that involved patients with this condition and found no effect of statins on incident AF. In conflict with these results, statin treatment has been associated with a 19% decrease of AF risk in patients ≥65 years old [16]. The decrease in AF risk was smaller in our analysis, perhaps due to differences in the studied populations. Hung et al. [16] reported that statin therapy was not as beneficial in patients without other cardiovascular comorbidities. We specifically excluded the population that might be taking statins for prevention of ischemic heart disease, defined as persons with history of ischemic heart disease, stroke, transient ischemic attack, and peripheral artery disease [17]. These exclusion criteria may imply higher risk of AF, and therefore our study population could be considered at lower risk of new-onset AF.

### Statin adverse effects

No excess of severe adverse effects related to statin initiation was found during follow-up. Increased incidence of diabetes associated with statin treatment has been evidenced in a meta-analysis of randomised controlled trials, although no statistical significance was shown in patients with hypertension [33], in accordance with our results. Diabetes [34], myopathy [35] and hepatopathy [36] are more frequent in intensive statin treatment regimes, and 88% of persons treated with statins were in low-moderate potency regimes in our study. In agreement with previous reports [37,38], we found no increased risk of cancer or hemorrhagic stroke associated with statin initiation. Still, we cannot exclude the possibility that incidence of diabetes, cancer, or haemorrhagic stroke might have increased in this population with longer statin exposure.

### Study characteristics and limitations

We focused on the study of a specific population of interest at increased risk of AF, i.e., hypertensive individuals with no ischemic heart disease, in response to the request for research on personalising the approach of patients at risk of this arrhythmia [2,13,39,40]. We had access to SIDIAP^Q^, a large, anonymised, high-quality dataset that includes a considerable number of participants with new-onset AF. Electronic medical records provide the opportunity to address certain questions related to the effect of medical treatments. They contain data on individuals often excluded from clinical trials (e.g. women, persons with diabetes), and thus reflect ‘real-world’ practice, at a reasonable cost [41], and there is evidence of a correspondence between studies based on electronic medical records and randomised controlled trials [42].

At the same time, the peril of biased results has been suggested for observational studies, unless some key points are addressed [43]. Concerning statins effects, study populations must be comparable such that they differ only in their statin use. Thus, in addition to using a new–users design (as opposed to prevalent users) we adjusted statin use by the propensity score (PS) for this treatment to prevent indication bias, and randomly allocated the index dates of statin non-users following the distribution of new-users to prevent immortality bias[44]. We also applied sample restriction, excluding patients with cancer, dementia, plegia, transplanted, institutionalized, or in dialysis, to reduce the healthy user bias [45, 46]. However, some unmeasured factors may influence prescription patterns and treatment adherence, including unreported side effects, frequency of access to medical care, and patient willingness to take the drug [47].

To avoid the selection bias associated with missing values, we replaced those of the continuous variables, instead of excluding these records. Individuals with missing values had a slightly healthier profile, and the process of multiple imputation was intended to account for this. The characteristics of the study population met plausibility criteria for the missing-at-random assumption for all imputed variables except the MEDEA deprivation index. Its missing mechanism was completely at random, thus exclusion of participants who lacked a MEDEA score did not imply selection bias.

We could not distinguish between various types of AF because we used ICD codes to identify the diagnosis. Similarly, we could not differentiate AF from atrial flutter, because they share the same ICD code. Underrecording of AF also could not be excluded, but if it occurred, it would have been randomly distributed among new-users and non-users, and any potential bias would have tended towards the null. Finally, underrecording of moderate myopathy and hepatopathy could partially explain the lack of association of these adverse effects with statins, since their low incidence hindered accurate estimation of their association with statin use.

## Conclusions

Statin initiation showed a statistically significant 9% reduction of new-onset AF that was not clinically relevant, as evidenced by a high 1-year NNT. Even in the highest risk group, the absolute risk reduction was too small to support the use of statins solely for primary prevention of incident AF. Furthermore, their adverse effects could not be unreservedly dismissed.

## Supporting information

S1 Appendix (Table A) Cox proportional hazards model predicting the risk of new-onset AF. (Table B) Observed and predicted rates of new-onset AF within 5-year risk groups. (Table C) Missing values and baseline characteristics by statin use and imputation.(Table D) Baseline characteristics by statin use and propensity score. Complete cases. (Table E) AF hazard ratios of statin use and its adverse effects. Complete cases.
(DOCX)
