# Supplementary material for: Statins and new-onset atrial fibrillation in a cohort of patients with hypertension. Analysis of electronic health records, 2006–2015
Source: PLoS One. 2017 Oct 26;12(10):e0186972. doi: 10.1371/journal.pone.0186972 (PMC5658105; doi:10.1371/journal.pone.0186972)
Supplement: S1 Appendix — (Table A) Cox proportional hazards model predicting the risk of new-onset AF. (Table B) Observed and predicted rates of new-onset AF within 5-year risk groups. (Table C) Missing values and baseline characteristics by statin use and imputation.(Table D) Baseline characteristics by statin use and propensity score. Complete cases. (Table E) AF hazard ratios of statin use and its adverse effects. Complete cases. (DOCX) [file pone.0186972.s001.docx]

**S1 APPENDIX**

Title

**Statins and new-onset atrial fibrillation in a cohort of patients with hypertension. Analysis of electronic health records, 2006-2015**

Authors

Lia Alves-Cabratosa^a^, MD, PhD; Maria García-Gil^a,b^, MD, PhD; Marc Comas-Cufí^a^, MSc; Anna Ponjoan^a,c^, PhD; Ruth Martí-Lluch^a,c^, PhD; Dídac Parramon^a,d^, MD; Jordi Blanch^a^, MSc; Rafel Ramos^a,b,d^, MD, PhD.

Affiliations

1. Vascular Health Research Group of Girona (ISV-Girona). Jordi Gol Institute for Primary Care Research (IDIAP Jordi Gol), Spain.
2. Translab Research Group. Department of Medical Sciences, School of Medicine, University of Girona, Spain.
3. Girona Biomedical Research Institute (IDIBGI), Dr. Trueta University Hospital, Spain.
4. Primary Care Services, Girona. Catalan Institute of Health (ICS), Spain.

Corresponding author

Dr. Rafel Ramos, Vascular Health Research Institute of Girona, Jordi Gol Institute for Primary Care Research.

Tel. + 34 972487968

Fax. + 34 972214100

E-mail: [rramos.girona.ics@gencat.cat](mailto:rramos.girona.ics@gencat.cat)

**Table of contents**

[Supplemental Tables 3](#_Toc496083400)

[**Table A. Cox proportional hazards model predicting the risk of new-onset AF** 3](#_Toc496083401)

[**Table B. Observed and predicted rates of new-onset AF within 5-year risk groups** 4](#_Toc496083402)

[Sensitivity analyses. Complete case [1] 5](#_Toc496083403)

[**Table C.** **Missing values and baseline characteristics by statin use and imputation** 5](#_Toc496083404)

[**Table D.** **Baseline characteristics by statin use and propensity score. Complete cases** 7](#_Toc496083405)

[**Table E.** **AF hazard ratios of statin use and its adverse effects. Complete cases** 10](#_Toc496083406)

[Statistical Appendix 11](#_Toc496083407)

[Estimating the five-year risk of new-onset AF among the 100276 participants in the study 11](#_Toc496083408)

[Separating study participants according to baseline 5-year major vascular event risk 11](#_Toc496083409)

[References 12](#_Toc496083410)

# Supplemental Tables

Table A specifies the hazard ratios (95% CI) of the multiadjusted model to estimate the risk of new-onset AF.

### **Table A. Cox proportional hazards model predicting the risk of new-onset AF**

| Variable | HR (95% CI)* | p value |
| --- | --- | --- |
| Age, y | 1.090 (1.087 - 1.092) | <0.0001 |
| Weight, kg | 1.023 (1.022 - 1.025) | <0.0001 |
| COPD | 1.501 (1.398 - 1.612) | <0.0001 |
| Heart failure | 1.833 (1.625 - 2.069) | <0.0001 |
| Valvular heart disease | 2.280 (2.030 - 2.560) | <0.0001 |

*Hazard ratios are shown per one unit of increase in continuous variables, and for the presence of the condition in dichotomous variables.

CI indicates confidence interval; COPD chronic obstructive pulmonary disease; HR, hazard ratio.

Table B shows how observed and predicted risks within each 5-year risk group compare well.

### **Table B. Observed and predicted rates of new-onset AF within 5-year risk groups**

| Risk categories | Number of participants | Median (1^st^ – 3^rd^ quartiles) follow-up | Observed AF incidence (%) (95% CI) | Average (SD) predicted AF risk (%) |
| --- | --- | --- | --- | --- |
| Total population | 100 276 | 8.5 (8.1- 8.9) | 6.3 (6.1-6.4) | 5.6 (5.1) |
| AF risk group |  |  |  |  |
| <2.5% | 28 566 | 8.7 (8.3- 9.0) | 1.8 (1.7-1.9) | 1.8 (0.4) |
| ≥2.5 to <7.5 | 49 058 | 8.6 (8.1- 9.0) | 5.7 (5.6-5.9) | 4.4 (1.4) |
| ≥7.5 | 22 652 | 8.1 (5.0- 8.8) | 14.6 (14.2-15.0) | 12.8 (6.1) |

AF indicates atrial fibrillation; CI, confidence interval; SD, standard deviation

## Sensitivity analyses. Complete case [1]

Table C shows the baseline characteristics of the complete case and the imputed datasets of new-users compared to non-users of statins. New-users had a lower percentage of missing values.

### **Table C.** **Missing values and baseline characteristics by statin use and imputation**

| Variable | New-users | Non-users |
| --- | --- | --- |
| Total N | 4367 | 24583 |
| Systolic blood pressure (mmHg) |  |  |
| Missing values–N (%) | 1788 (18.2) | 26547 (29.4) |
| Complete cases –mean (SD) | 139.0 (15.7) | 137.7 (15.3) |
| Imputed –mean (SD) | 138.5 (16.3) | 137.6 (16.3) |
| Diastolic blood pressure (mmHg) |  |  |
| Missing values –N (%) | 1882 (19.2) | 27196 (30.1) |
| Complete cases –mean (SD) | 79.2 (9.2) | 78.5 (9.1) |
| Imputed –mean (SD) | 79.4 (9.5) | 78.9 (9.5) |
| Pulse pressure (mmHg) |  |  |
| Missing values –N (%) | 1883 (19.2) | 27246 (30.1) |
| Complete cases –mean (SD) | 59.7 (14.1) | 59.2 (14.0) |
| Imputed –mean (SD) | 59.1 (14.4) | 58.8 (14.6) |
| Weight (Kg) |  |  |
| Missing values –N (%) | 3798 (38.7) | 43573 (48.2) |
| Complete cases–mean (SD) | 76.1 (13.2) | 75.3 (13.4) |
| Imputed –mean (SD) | 75.6 (13.4) | 74.6 (13.4) |
| Height (cm) |  |  |
| Missing values –N (%) | 668 (6.8) | 8101 (9.0) |
| Complete cases –mean (SD) | 158.3 (8.8) | 158.2 (8.9) |
| Imputed –mean (SD) | 158.7 (8.9) | 158.9 (9.1) |
| BMI (kg/m^2^) |  |  |
| Missing values –N (%) | 3821 (38.9) | 43786 (48.4) |
| Complete cases –mean (SD) | 30.3 (4.8) | 30.0 (4.9) |
| Imputed –mean (SD) | 30.0 (4.8) | 29.5 (4.8) |
| Glycaemia (mg/dl) |  |  |
| Missing values –N (%) | 2585 (26.3) | 42410 (46.9) |
| Complete cases –mean (SD) | 115.1 (38.2) | 107.6 (32.2) |
| Imputed –mean (SD) | 110.2 (34.8) | 102.4 (26.4) |
| Total cholesterol (mg/dl) |  |  |
| Missing values –N (%) | 2542 (25.9) | 42968 (47.5) |
| Complete cases –mean (SD) | 246.9 (39.7) | 211.3 (32.7) |
| Imputed –mean (SD) | 245.0 (40.5) | 209.8 (32.6) |
| HDL cholesterol (mg/dl) |  |  |
| Missing values –N (%) | 3159 (32.2) | 52220 (57.7) |
| Complete cases–mean (SD) | 56.3 (14.1) | 57.4 (14.5) |
| Imputed –mean (SD) | 56.6 (14.0) | 57.7 (14.2) |
| LDL cholesterol (mg/dl) |  |  |
| Missing values –N (%) | 3165 (32.3) | 52384 (57.9) |
| Complete cases –mean (SD) | 160.5 (35.0) | 130.2 (28.7) |
| Imputed –mean (SD) | 159.3 (36.2) | 129.1 (28.8) |
| Triglycerides (mg/dl) |  |  |
| Missing values –N (%) | 2961 (30.2) | 50009 (55.3) |
| Complete cases –mean (SD) | 150.3 (83.5) | 119.5 (61.1) |
| Imputed –mean (SD) | 145.8 (84.9) | 115.2 (57.3) |

BMI indicates body mass index; HDL, High density lipoprotein; LDL, low density lipoprotein; N, number of participants with missing values in any of the imputed variables; SD, standard deviation.

Table D details baseline characteristics comparing statin new-users and non-users within the complete case dataset, that is, excluding participants with missing values. Participants in the complete case dataset present slightly higher percentage of diabetes and slightly higher prevalence of the considered comorbidities and concomitant treatments.

### **Table D.** **Baseline characteristics by statin use and propensity score. Complete cases**

|  | Before PS adjustment | | | | After PS adjustment | | |
| --- | --- | --- | --- | --- | --- | --- | --- |
|  | New-users  (n=4367) | | Non-users (n=24583) | SDf | New-users (n=4367) | Non-users (n=24583) | SDf |
| Age, years-* | 67.6 (7.7) | | 68.6 (8.3) | 0.13 | 68.5 (7.7) | 68.5 (8.3) | <0.01 |
| Men | 38.2 | | 37.5 | -0.01 | 37.5 | 37.6 | <0.01 |
| MEDEA index |  | |  |  |  |  |  |
| Rural areas | 13.74 | | 17.50 | 0.10 | 17.11 | 16.47 | -0.02 |
| Urban areas (quintile) |  | |  |  |  |  |  |
| First | 4.5 | | 4.6 | <0.01 | 4.6 | 4.6 | <0.01 |
| Second | 12.2 | | 12.3 | <0.01 | 12.0 | 12.3 | 0.01 |
| Third | 21.4 | | 21.0 | -0.01 | 20.8 | 21.1 | 0.01 |
| Fourth | 23.6 | | 22.5 | -0.03 | 22.4 | 22.6 | 0.01 |
| Fifth | 24.6 | | 22.2 | -0.06 | 22.3 | 22.5 | 0.01 |
| Systolic BP, mmHg* | 139 (15.7) | | 137.7 (15.3) | -0.08 | 138 (15.6) | 137.9 (15.3) | -0.01 |
| Diastolic BP, mmHg* | 79.2 (9.2) | | 78.5 (9.1) | -0.08 | 78.6 (9.1) | 78.6 (9.1) | -0.01 |
| Pulse pressure, mmHg* | 59.7 (14.1) | | 59.2 (14) | -0.04 | 59.4 (14.1) | 59.3 (14) | -0.01 |
| Weight, Kg* | 76.1 (13.2) | | 75.3 (13.4) | -0.06 | 75.3 (13.2) | 75.5 (13.4) | 0.01 |
| Height, cm* | 158.3 (8.8) | | 158.2 (8.9) | -0.01 | 158.2 (8.8) | 158.2 (8.9) | <0.01 |
| BMI, Kg/m^2^* | 30.3 (4.8) | | 30 (4.8) | -0.07 | 30 (4.8) | 30 (4.8) | 0.01 |
| Total cholesterol, mg/dl* | 246.9 (39.7) | | 211.3 (32.7) | -0.98 | 216.9 (20.9) | 216.7 (19.6) | -0.01 |
| HDL-c, mg/dl* | 56.3 (14.1) | | 57.4 (14.5) | 0.07 | 57.2 (14.1) | 57.2 (14.5) | <0.01 |
| LDL-c, mg/dl* | 160.5 (35) | | 130.2 (28.7) | -0.95 | 134.7 (20.4) | 134.8 (17.6) | 0.01 |
| Triglycerides, mg/dl* | 150.3 (83.5) | | 119.5 (61.1) | -0.42 | 126.3 (78.5) | 123.8 (57.6) | -0.04 |
| Glucose, mg/dl-* | 115.1 (38.2) | | 107.6 (32.2) | -0.21 | 108.9 (37.6) | 108.7 (31.7) | -0.01 |
| Alcohol consumption |  | |  |  |  |  |  |
| No | 90.2 | | 90.9 | 0.02 | 89.7 | 91.0 | 0.04 |
| Low-risk | 8.8 | | 8.3 | -0.02 | 9.3 | 8.2 | -0.04 |
| High risk | 1.0 | | 0.8 | -0.02 | 0.9 | 0.8 | -0.02 |
| Smokers | 19.6 | | 16.9 | -0.07 | 17.0 | 17.2 | 0.01 |
| Diabetes | 31.6 | | 23.4 | -0.18 | 23.1 | 24.1 | 0.02 |
| Arthritis | 1.1 | | 0.9 | -0.02 | 0.9 | 0.9 | <0.01 |
| Hyperthyroidism | 0.9 | | 1.0 | 0.02 | 1.0 | 1.0 | <0.01 |
| Hypothyroidism | 5.5 | | 5.7 | 0.01 | 5.6 | 5.6 | <0.01 |
| COPD | 7.0 | | 6.8 | -0.01 | 7.1 | 6.8 | -0.01 |
| Asthma | 4.2 | | 4.4 | 0.01 | 4.4 | 4.4 | <0.01 |
| Sleep apnoea | 1.8 | | 1.4 | -0.03 | 1.4 | 1.5 | <0.01 |
| Chronic kidney disease | 2.4 | | 2.0 | -0.02 | 2.1 | 2.1 | <0.01 |
| Valvular heart disease | 2.1 | | 1.9 | -0.01 | 1.9 | 1.9 | <0.01 |
| Heart failure | 0.8 | | 1.0 | 0.02 | 0.9 | 0.9 | <0.01 |
| Concomitant treatment |  | |  |  |  |  |  |
| Diuretics | 34.7 | | 32.9 | -0.04 | 33.1 | 33.2 | <0.01 |
| Beta blockers | 15.5 | | 13.8 | -0.05 | 14.1 | 13.9 | <0.01 |
| Calcium channel blockers ** | 13.4 | | 11.2 | -0.07 | 11.0 | 11.4 | 0.01 |
| Agents acting on renin-angiotensin system | 64.0 | | 57.2 | -0.14 | 57.3 | 58.6 | 0.03 |
| Other antihypertensives | 3.2 | | 3.7 | 0.03 | 3.5 | 3.6 | 0.01 |
| Hypoglycaemic agents | 23.1 | | 16.0 | -0.18 | 15.2 | 16.3 | 0.03 |
| Lipid-lowering drugs, non-statins | 5.7 | | 3.3 | -0.12 | 2.9 | 3.2 | 0.02 |
| AF risk subgroups | |  |  |  |  |  |  |
| <2,5% | | 26.3 | 24.0 | -0.05 | 24.2 | 24.3 | <0.01 |
| ≥2.5-7.5% | | 53.7 | 52.0 | -0.03 | 52.9 | 52.1 | -0.01 |
| ≥7.5% | | 20.0 | 24.0 | 0.10 | 22.5 | 23.4 | 0.02 |
| Framingham-REGICOR risk† | | 7.3 (5.7) | 5.6 (4.7) | -0.32 | 6 (5.6) | 5.8 (4.6) | -0.04 |
| Framingham-REGICOR <10% risk (subgroups, %)‡ | |  |  |  |  |  |  |
| AF risk <2,5% | | 85.1 | 94.7 | 0.32 | 94.9 | 95.1 | 0.01 |
| AF risk ≥2.5-7.5% | | 75.7 | 87.4 | 0.31 | 88.1 | 87.8 | -0.01 |
| AF risk ≥7.5% | | 68.7 | 75.6 | 0.16 | 77.8 | 75.3 | -0.06 |

Data are presented as % unless otherwise indicated.

*Shown as mean (SD).

** Selective calcium channel blockers with mainly vascular effects.

† Framingham-REGICOR coronary risk function.

‡Among those with a score >10% on the Framingham-REGICOR coronary risk function [2], % of participants within each AF risk subgroup.

BMI indicates body mass index; BP, blood pressure; COPD, chronic obstructive pulmonary disease; HDL-c, high density lipoprotein cholesterol; LDL-c, low density lipoprotein cholesterol; MEDEA, socioeconomic deprivation index; N, number of cases; PS, propensity score of statin treatment; SD, standard deviation; SDf, standardised differences.

Table E shows the hazard ratios HRs (95% confidence interval –CI- ) of being a new-user of statins, the number needed to treat to avoid one case of new-onset AF in one year, and additional characteristics of interest with regard to AF incidence in statin new-users and non-users, amongst all the population, and within each risk group. These results do not substantially differ from those with imputed data, except for the slightly higher AF incidence in non-users in the imputed dataset compared to the complete-case dataset (as stated in the main Results section, Sensitivity analyses). The incidences of the adverse effects considered are comparable to those in the imputed dataset. The diabetes incidence rate is higher in both new-users and non-users in the complete case analysis.

### **Table E.** **AF hazard ratios of statin use and its adverse effects. Complete cases**

|  | New-users | |  | Non-users | |  |
| --- | --- | --- | --- | --- | --- | --- |
|  | Events | Incidence rate* (95% CI) |  | Events | Incidence rate* (95% CI) | HR (95%CI) |
| **AF, total population** | 385 | 10.9 (9.8-12.0) |  | 2520 | 13.0 (12.5-13.5) | 0.94 (0.83-1.06) |
| **AF risk groups** |  |  |  |  |  |  |
| <2.5% | 27 | 2.7 (1.7-3.8) |  | 169 | 3.4 (2.9-3.9) | 0.80 (0.51-1.26) |
| ≥2.5 to <7.5 | 192 | 10.0 (8.6-11.4) |  | 1178 | 11.4 (10.8-12.1) | 0.97 (0.82-1.14) |
| ≥7.5 | 166 | 26.2 (22.2-30.2) |  | 1173 | 28.2 (26.6-29.8) | 1.04 (0.87-1.24) |
| **Adverse effects** | | | | | | |
| Cancer | 653 | 21.9 (20.3-23.6) |  | 3798 | 23.1 (22.4-23.9) | 0.99 (0.90-1.09) |
| Haemorrhagic stroke | 59 | 1.9 (1.4-2.3) |  | 349 | 2.0 (1.7-2.2) | 0.96 (0.70-1.31) |
| Diabetes | 1123 | 43.1 (40.6-45.6) |  | 5437 | 35.9 (35.0-36.9) | 0.98 (0.91-1.06) |
| Hepatotoxicity | 3 | - |  | 15 | 0.6 (0.3-0.9) | - |
| Myopathy | 1 | - |  | 5 | 0.2 (0.03-0.4) | - |

*per 1000 person-year

†at 1 year

AF indicates atrial fibrillation; CI, confidence interval; HR, hazard ratio.

# Statistical Appendix

## Estimating the five-year risk of new-onset AF among the 100276 participants in the study

The final equation to estimate the 5-year individual risk of new-onset AF originates from the expression for the proportional hazards risk function allowing for more than one covariate:

R(t, x, β)=1-[S_0_(t)]^exp(x’ β)^

where

R(t, x, β) is the Risk at a time t, depending on specific covariates x and their estimated coefficients β. S_0_(t) is the baseline survival function at a time t [3].

The risk function, R(t), depending on age, weight, COPD, heart failure, and valvular heart disease, for new-onset AF is:

R ( t = 5, age, weight, COPD, heart failure, valvular heart disease)=1−(0.9999789929)exp(y)

where

y = 0.0858 age (years) + 0.0231 weight (kg) + 0.4064 COPD (yes/no) + 0.6062 heart failure (yes/no) + 0.8241 valvular heart disease (yes/no)

For this Cox proportional hazard model, candidate variables were selected using forward-backward elimination in bootstrap models, with factors being included in the final model if they remained in 100% of the preliminary bootstrap models.

## Separating study participants according to baseline 5-year major vascular event risk

Study participants were categorised into one of three baseline categories of 5-year risk: <2.5%; ≥2.5 to 7.5%; ≥7.5%. The absolute and relative effects of statin treatment on new-onset AF was then estimated separately within each of these subgroups (as described in the main Methods section –in Variable Definition, Exposure).

# References

1 Sterne J a C, White IR, Carlin JB, Spratt M, Royston P, Kenward MG, *et al.* Multiple imputation for missing data in epidemiological and clinical research: potential and pitfalls. *BMJ* 2009; 338:b2393–b2393.

2 Marrugat J, Subirana I, Comín E, Cabezas C, Vila J, Elosua R, *et al.* Validity of an adaptation of the Framingham cardiovascular risk function: the VERIFICA Study. *J Epidemiol Community Health* 2007; 61:40–7.

3 Hosmer DW, Lemeshow S, May S. *Applied Survival Analysis*. 2nd ed. Hoboken, NJ: Wiley; 2008.
